# Supplementary material for: Hydrogenation of iron in the early stage of Earth's evolution
Source: Nat Commun. 2017 Jan 13;8:14096. doi: 10.1038/ncomms14096 (PMC5241797; doi:10.1038/ncomms14096)
Supplement: Supplementary Information — Supplementary Figures and Supplementary Tables [file ncomms14096-s1.pdf]

Supplementary Information

Supplementary figures

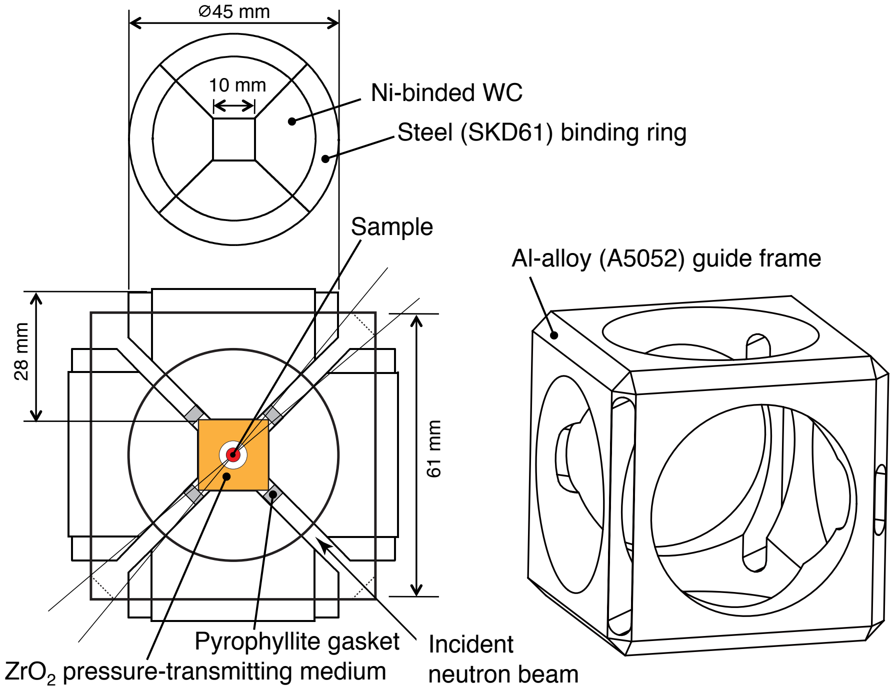

Supplementary Figure 1 | Experimental setup. Newly designed anvil assembly with 6-6-type multi-anvil cells.

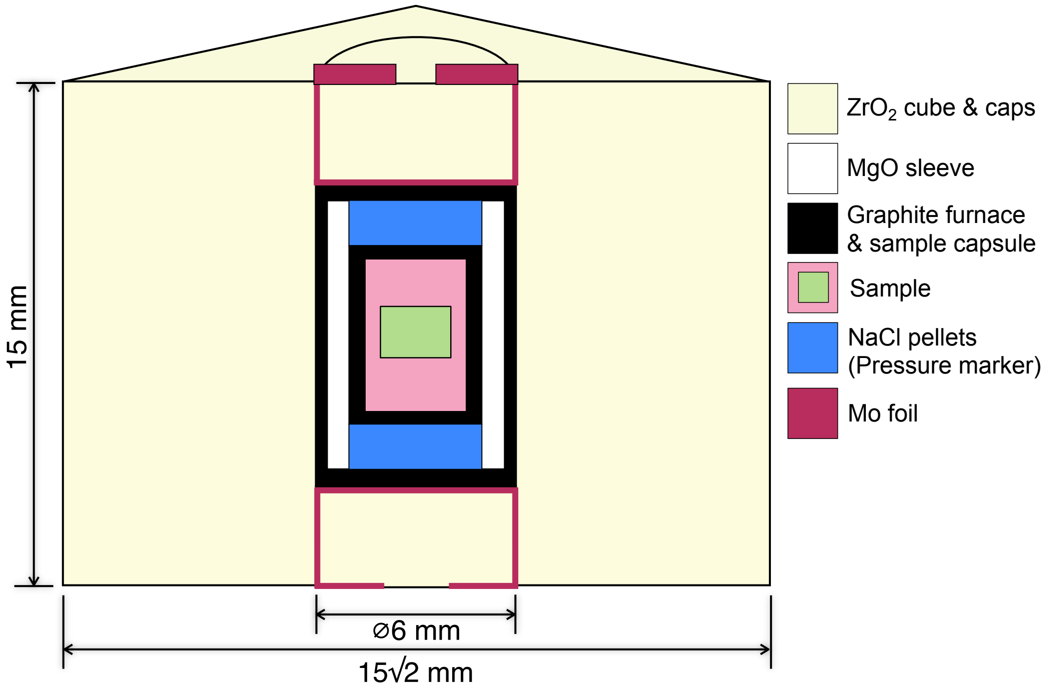

Supplementary Figure 2 | Experimental setup. Cross-section of the cell assembly.

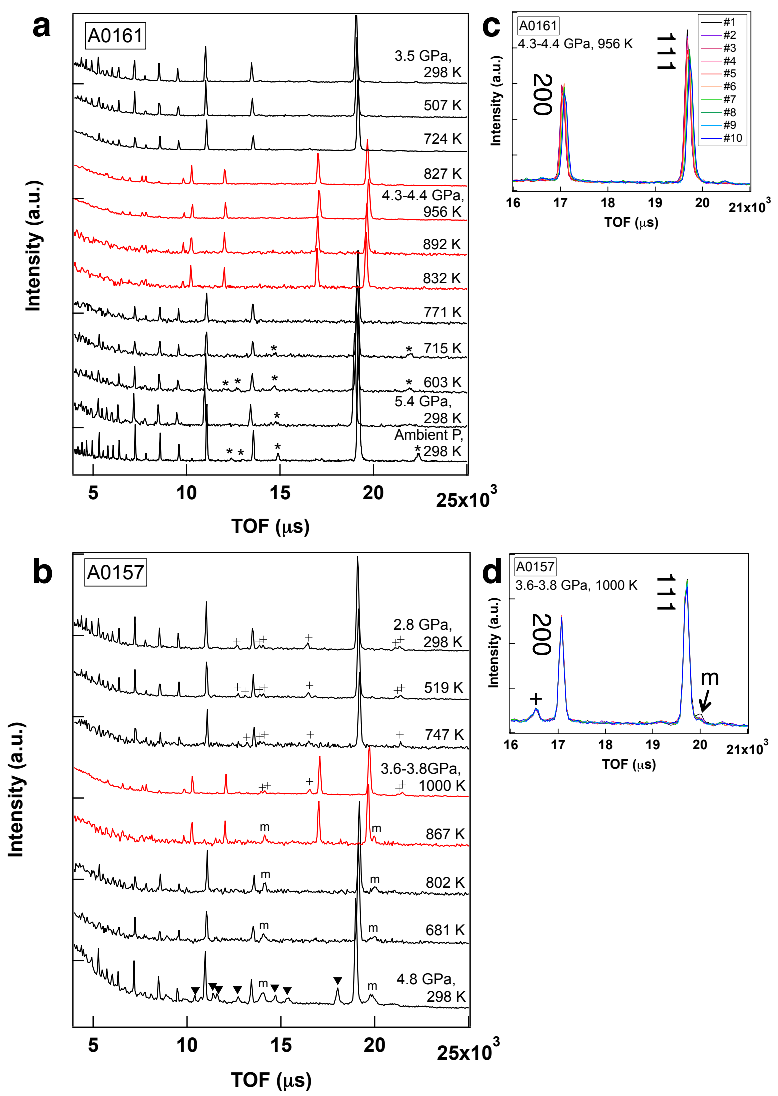

Supplementary Figure 3 | Entire dataset of powder neutron diffraction patterns obtained in runs (a) #A0161 and (b) #A0157. The series of observations was made from bottom to top. The red patterns show diffractions when iron or its hydride was in an *fcc* structure. In #A0161 $Mg(OD)_2$ (marked with "*") was observed before dehydration occurred, as in #A0156 (Figure 1) while in #A0157 MgO (marked with "m") was observed. Both of hem disappeared after olivine (marked with "+") was formed. Data at 4.8 GPa and 298 K in #A0157 was obtained using wide slit (1.0w x 5.3h) to get diffractions from NaCl pressure marker (marked with "▼"). The two strong diffraction peaks of *fcc* iron or its hydride, 111 and 200, measured every hour for ~10 hours, are shown on the right figures, **(c)** and **(d)**. Note that all the peaks for #A0157 completely overlap, indicating that there were no changes in both the positions and the line width, while the peaks for #A0161 shifted slightly to the right with time, without changing the line width.

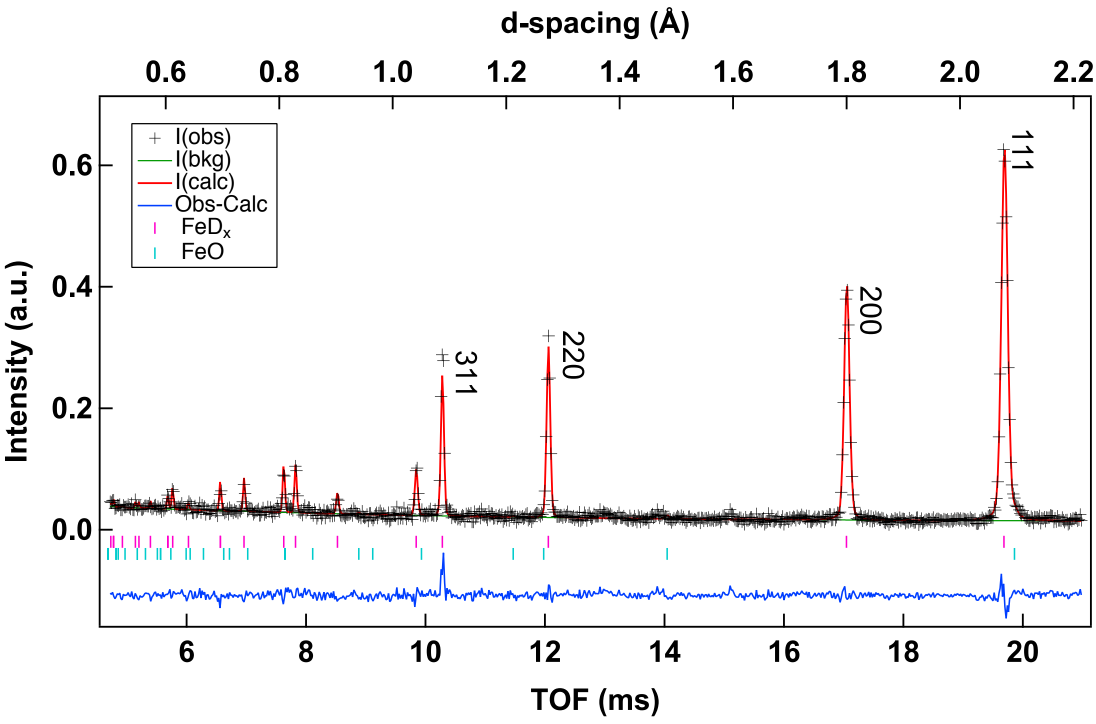

Supplementary Figure 4 | Results of the Rietveld analysis for one hour data collected in #A0161. "+" denotes observed data points; the solid red line denotes the calculated profile. Tick marks below the pattern show the positions of diffraction peaks calculated for FeD_x (upper row) and FeO (lower row). The difference between the observed and calculated profiles is shown at the bottom.

Supplementary Tables

Supplementary Table 1 | Summary of experimental conditions and results.

Run#	*P* max (kN / GPa)	*T* max (K)	Starting material	Products
A0161	1200 / 5.4	956	Fe + $Mg(OD)_2$ + SiO_2	Fe, FeO, olivine, pyroxene
A0156	1000 / 4.9	1000	Fe + $Mg(OD)_2$ + SiO_2	Fe, FeO, olivine, pyroxene
A0157	1000 / 4.8	1000	Fe + MgO + SiO_2	Fe, olivine, pyroxene

Supplementary Table 2 | Results of volume change and deuteration from Rietveld analysis measured during a long holding time. Notes: *Elapsed time since the start of measurements after holding at the maximum temperature; †Pressures calculated from NaCl, for which data were obtained shortly before the sample measurements, are given (see also Fig. 2); ‡Each zero-volume (V_0) was taken from the initial value of the long holding time. Errors were estimated based on the standard deviation of Rietveld analysis. Values for #A0157 under dry condition are too low to be meaningful. g_D(O) and g_D(T) represent site occupancies of deuterium in the O and T sites, respectively. U_{iso} is the isotropic atomic displacement parameter, constrained to be identical for the same D species. Deuterium content is denoted by *x*.

Run#	Data#	Time* (hh:mm:ss)	*P*† (GPa)	*T* (K)	*a* (Å)	*V* (Å^3)	*ΔV*‡ (%)	gD(O)	gD(T)	U_{iso}_Fe (100 x Å^2)	U_{iso}_D (100 x Å^2)	R_{wp} (%)	R_p (%)	*x*	*x* (wt.%)
A0161	16625	0:36:14	4.3	956	3.6011(1)	46.697(5)	0	0.116(12)	0.018(10)	1.1	4.5	14.1	11.1	0.15(2)	0.54(3)
	16626	1:47:05			3.6012(1)	46.701(4)	0.01(1)	0.147(10)	0.039(9)	1.2	5.0	12.0	10.3	0.23(2)	0.81(2)
	16627	2:56:15			3.6012(1)	46.727(4)	0.06(1)	0.130(11)	0.017(10)	1.1	5.1	13.4	11.4	0.16(2)	0.58(3)
	16629	4:30:22	4.4		3.6030(1)	46.771(4)	0.16(1)	0.118(10)	0.006(9)	1.1	3.3	12.8	11.0	0.13(2)	0.46(2)
	16630	5:39:15			3.6040(1)	46.812(4)	0.25(1)	0.132(11)	0.016(10)	1.1	5.2	13.5	11.3	0.16(2)	0.58(3)
	16631	6:49:20			3.6056(1)	46.875(4)	0.38(1)	0.137(11)	0.013(10)	1.2	4.6	13.0	11.3	0.16(2)	0.59(3)
	16633	8:23:44	4.4		3.6098(1)	47.040(5)	0.73(2)	0.194(12)	0.037(12)	1.4	6.3	13.9	11.7	0.27(2)	0.96(3)
	16634	9:43:43			3.6117(1)	47.112(5)	0.89(2)	0.157(10)	0.030(10)	1.2	4.4	15.6	11.9	0.22(2)	0.77(3)
	16635	10:59:17			3.6120(1)	47.126(5)	0.92(2)	0.149(10)	0.029(9)	1.2	4.1	11.7	10.8	0.21(2)	0.74(2)
	16637	12:33:37	4.3		3.6126(1)	47.147(5)	0.96(2)	0.142(10)	0.011(9)	1.1	3.9	13.5	11.2	0.16(2)	0.59(2)

Supplementary Table 2 (continued)

Run#	Data#	Time * (hh:mm:ss)	P † (GPa)	T (K)	a (Å)	V (Å^3)	ΔV ‡ (%)	gD(O)	gD(T)	U_{iso}_Fe (100 x Å^2)	U_{iso}_D (100 x Å^2)	R_{wp} (%)	R_p (%)	x	x (wt.%)
A0156	16473	0:31:35	3.9	1000	3.6070(1)	46.929(4)	0	0.125(9)	0.024(8)	1.2	3.9	12.0	10.5	0.17(2)	0.62(2)
	16474	1:41:57			3.6080(1)	46.965(4)	0.08(1)	0.101(11)	0.003(9)	1.2	3.7	11.6	9.60	0.18(2)	0.66(3)
	16475	2:49:55			3.6089(1)	47.005(4)	0.16(1)	0.130(11)	0.014(10)	1.3	5.2	12.8	10.3	0.16(2)	0.56(3)
	16476	3:57:52			3.6104(1)	47.061(4)	0.28(1)	0.125(9)	0.011(8)	1.2	4.9	11.0	9.67	0.15(2)	0.52(2)
	16477	5:07:19			3.6138(1)	47.195(4)	0.57(1)	0.132(9)	0.016(8)	1.3	4.2	11.7	9.61	0.16(2)	0.58(2)
	16478	6:17:17			3.6156(1)	47.263(4)	0.71(1)	0.150(9)	0.013(8)	1.3	3.3	11.3	10.2	0.17(2)	0.62(2)
	16479	7:25:16			3.6161(1)	47.285(5)	0.76(1)	0.149(10)	0.016(9)	1.2	4.2	12.3	10.8	0.18(2)	0.64(2)
	16480	8:33:13			3.6161(1)	47.285(5)	0.76(1)	0.138(10)	0.010(9)	1.2	5.2	12.6	10.5	0.16(2)	0.56(2)
	16481	9:43:25			3.6169(1)	47.318(4)	0.83(1)	0.149(10)	0.021(9)	1.3	4.1	11.4	10.6	0.19(2)	0.68(2)
A0157	16547	0:23:22	3.8	1000	3.6073(1)	46.940(5)	-	0.110(11)	-	2.2	7.2	17.0	12.0	0.11(1)	0.39(2)
	16548	1:31:26			3.6069(1)	46.925(5)	-	0.125(11)	0.019(10)	1.2	3.1	18.9	12.5	0.16(2)	0.58(3)
	16549	2:39:31			3.6066(1)	46.915(5)	-	0.10(2)	-	2.0	6.8	13.1	11.7	0.07(4)	0.26(5)
	16551	4:10:39	3.7		3.6066(1)	46.915(5)	-	0.139(13)	0.011(13)	1.2	6.7	17.0	13.2	0.16(0)	0.58(3)
	16552	5:47:18			3.6066(1)	46.918(6)	-	0.145(14)	0.011(12)	1.3	3.4	24.2	16.5	0.17(3)	0.59(3)
	16553	6:56:22			3.6067(1)	46.917(6)	-	0.135(14)	0.015(13)	1.3	4.1	22.1	15.4	0.17(3)	0.59(3)
	16555	8:30:44	3.6		3.6066(1)	46.914(6)	-	0.136(14)	0.014(13)	1.3	4.2	17.9	14.1	0.16(3)	0.58(3)
	16556	9:45:23			3.6065(1)	46.911(5)	-	0.114(9)	-	1.2	4.7	13.7	11.9	0.11(1)	0.41(2)
			3.7												
